# Different Parasite Faunas in Sympatric Populations of Sister Hedgehog Species in a Secondary Contact Zone

**DOI:** 10.1371/journal.pone.0114030

**Published:** 2014-12-03

**Authors:** Miriam Pfäffle, Barbora Černá Bolfíková, Pavel Hulva, Trevor Petney

**Affiliations:** 1 Department of Ecology and Parasitology, Zoological Institute, Karlsruhe Institute of Technology, Karlsruhe, Germany; 2 Faculty of Tropical AgriSciences, Czech University of Life Sciences Prague, Prague, Czech Republic; 3 Department of Zoology, Faculty of Science, Charles University Prague, Prague, Czech Republic; 4 Department of Biology and Ecology, Life Science Research Centre, University of Ostrava, Ostrava, Czech Republic; Field Museum of Natural History, United States of America

## Abstract

Providing descriptive data on parasite diversity and load in sister species is a first step in addressing the role of host-parasite coevolution in the speciation process. In this study we compare the parasite faunas of the closely related hedgehog species *Erinaceus europaeus* and *E. roumanicus* from the Czech Republic where both occur in limited sympatry. We examined 109 hedgehogs from 21 localities within this secondary contact zone. Three species of ectoparasites and nine species of endoparasites were recorded. Significantly higher abundances and prevalences were found for *Capillaria* spp. and *Brachylaemus erinacei* in *E. europaeus* compared to *E. roumanicus* and higher mean infection rates and prevalences for *Hymenolepis erinacei*, *Physaloptera clausa* and *Nephridiorhynchus major* in *E. roumanicus* compared to *E. europaeus*. Divergence in the composition of the parasite fauna, except for *Capillaria* spp., which seem to be very unspecific, may be related to the complicated demography of their hosts connected with Pleistocene climate oscillations and consequent range dynamics. The fact that all parasite species with different abundances in *E. europaeus* and *E. roumanicus* belong to intestinal forms indicates a possible diversification of trophic niches between both sister hedgehog species.

## Introduction

The restriction of populations of a wide variety of European species to spatially limited refuge areas during the cyclic climatic changes of the Pleistocene, together with the associated genetic bottleneck, has had a great impact on the genetic characteristics of the species and on speciation [1]. Many of the vertebrate species so affected harbor a specific parasite community which undergoes the same cyclic population, spatial and genetic restrictions, potentially leading to community changes, loss of genetic diversity and speciation [2], [3].

Hedgehogs were repeatedly restricted to glacial refuges during ice age maxima with subsequent re-colonization of Europe [1], [4]. Recent Western European hedgehogs (*Erinaceus europaeus*, *EE*) had a disjunct distribution on the Iberian Peninsula and Italy (Apennine and Sicilian refuges), while the northern white-breasted hedgehog (*E. roumanicus*, *ER*) survived in the Balkan refuge and the southern white-breasted hedgehog (*E. concolor*, *EC*) was found in the Middle East, separated from *ER* by the Bosporus and the Caucasus Mountains [5], [6].

Until recently *ER* was considered to belong either to *EE* or to *EC* and it has only recently been defined as a valid species [7]. Studies based on mitochondrial and nuclear sequence data suggest the sister position of *ER* and *EC* with a divergence time of approximately 1–2 Myr, [4], [8], [9]. Bannikova *et al.* [9] also showed the sister status of *EE* and *Erinaceus amurensis* (*EA*) with the time of separation being estimated as approximately 1 Myr. These two groups – *EE* + *EA* and *ER* + *EC* probably split during the Pliocene [9]. The secondary contact between *ER* and *EE* in central Europe probably originated after the last ice age during the Neolithic deforestation [10]. The distribution of these species is parapatric, however, the zone of overlap in central Europe reaches its greatest within the Czech Republic [10], [11]. Until now, no interspecific hybridization in the area of Central Europe has been recorded [8], [10], although Bogdanov *et al.* [12] reported a possible hybrid individual from Russia where the contact zone is younger.

**Figure 1:**
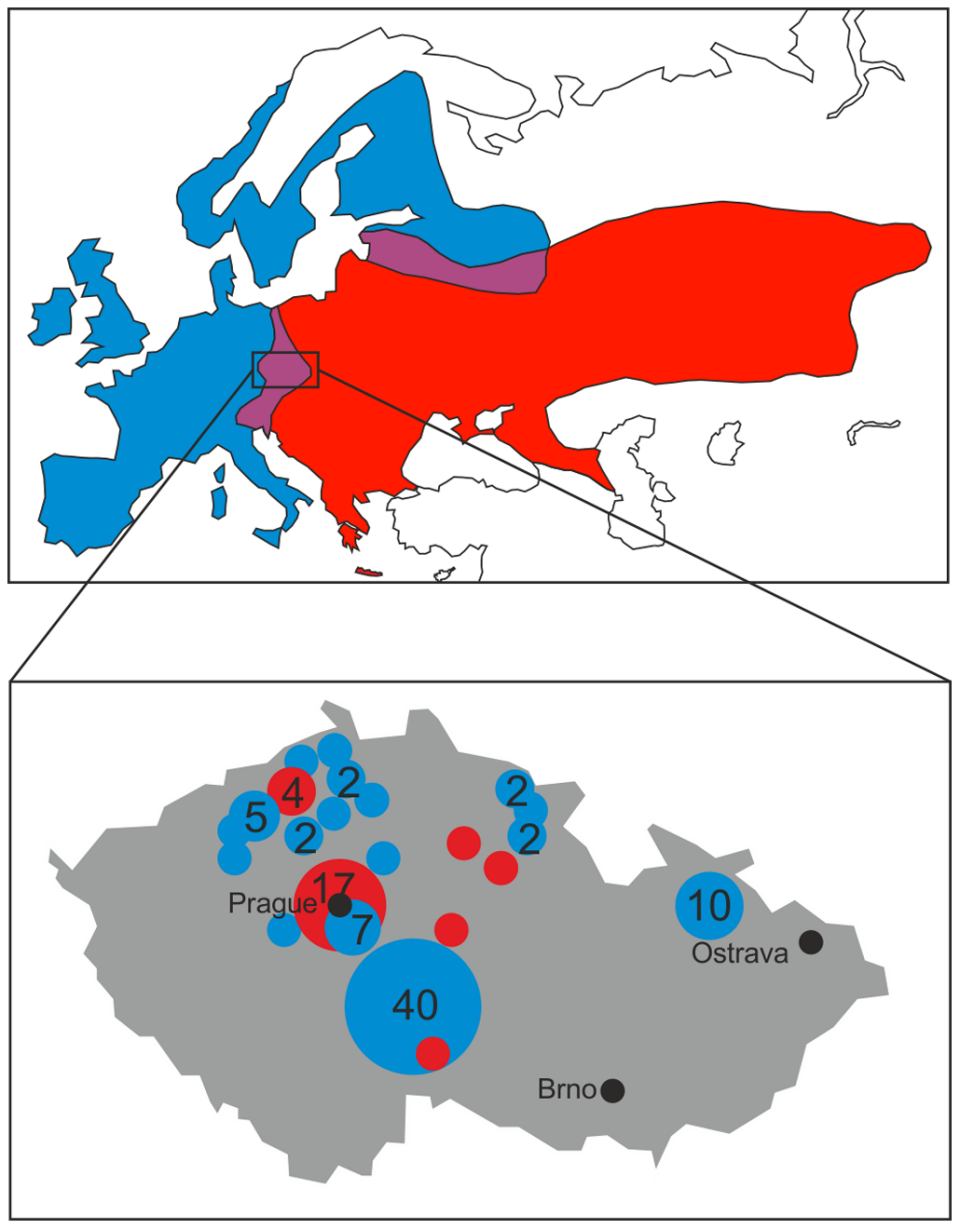
Distribution range map of *Erinaceus europaeus* (blue) and *E. roumanicus* (red) in the western Palearctic and their zones of sympatry (violet) as well as the origins of the hedgehog specimen from the Czech Republic used in this study. Size of circles represents the number of hedgehogs from the different locations (modified after [10]).

Currently, the Western Palearctic is inhabited by all three hedgehog species. *EC* ranges from Asia Minor to Israel, Syria, Lebanon, Iraq and Iran; and the southern Caucasus [7]. *ER* has a distribution extending from Central and Eastern Europe, the Baltic and the Balkan Peninsula, the Greek Adriatic including Rhodes and eastwards through Belarus, the Ukraine, and Russia, reaching as far as the Ob River in Siberia. In the south, its range extends as far as the northern Caucasus and the island of Crete. Within the Mediterranean region, it ranges from Italy and Slovenia, through the Balkan Peninsula, extending south into Thracian (i.e. European) Turkey [7]. These two species are separated in the Caucasus Mountains, but information about a contact zone is missing. The distribution of *EE* extends from the British Isles and the Iberian peninsula, westwards through much of western to central Europe; and from southern Fennoscandia, and the northern Baltic to north-west Russia. In the Mediterranean, it occurs in Portugal, France (including Corsica), Spain and Italy including Sardinia and Sicily [7]. In central Europe and in Russia and Estonia, the range of *EE* overlaps with that of *ER*. Figure 1 shows the distribution of *EE* and *ER* as well as their contact zones in the western Palearctic.

Hedgehogs host a wide variety of different macroparasites, endoparasitic helminths and ectoparasitic ticks, fleas and mites [13], with many widely distributed species but also some species which are narrowly endemic, such as *Brachylecithum mackoi* from the island of Elba [14]. These may play an important role in host morbidity and mortality (see e.g. [15], [16]). However, very little is known about the similarities and differences in the parasite fauna of the three Eurasian hedgehog species. Given the relatively deep split defining these hedgehog species, as well as the cyclic restriction in distributional area and population size, we predict that parasite co-speciation may have occurred and that the parasite community infesting the particular host species may differ. Thus, investigating the composition of the parasite fauna may provide valuable information regarding the phylogenetic differentiation, niche and population dynamics, diversification and species interactions in this group.

In this study we compare the parasite faunas of *ER* and *EE* from contact zones in the Czech Republic and interpret this information with regard to the phylogeography of the genus.

## Material and Methods

The hedgehogs from this study were either provided by wildlife rescue centers, where the animals died naturally, or were collected as road kills. Therefore an ethical approval by the relevant ethics committee was not required. All specimens were frozen at −20°C until they were used for dissection. Prior to examination individuals were thawed at room temperature, weighed and the sex was determined. Animals were classified either as hoglets (<100 g), juveniles (<500 g) or adults (>500 g) [16].

The samples originated from 21 different areas in the Czech Republic (Figure 1, Table S1). Collections were made during 2008–2011. In total we examined 109 animals (*ER*  = 27, *EE*  = 82). For *ER* we collected 19 juveniles (female  = 11, male  = 8) and seven adults (female  = 2, male  = 5). For *EE* we found eight hoglets (female  = 3, male  = 5), 61 juveniles (female  = 30, male  = 31) and 12 adults (female  = 5, male  = 7). Genetic analysis of mitochondrial DNA and nucelar microsatellites were used for determination of 20 specimens of *EE* and 14 specimens of *ER*, which were identical with the study of Bolfíková & Hulva [10]. Considering the presumed absence of hybridization (or very low degree of introgression) between both species in Central Europe ascertained by reciprocal monophyly of respective clades and the absence of intermediate phenotypes in the analysis of more than 200 individuals within the contact zone [10], morphology-based discrimination was used for remaining specimens within the present study.

Fleas and ticks were collected, identified to life history stage, sex (if not immature) as well as to species after Beaucournou & Launay [17] and Arthur [18], respectively, and subsequently quantified. Due to the difficulty of quantifying infestation rates, mites were not included in the examination. The body cavity (peritoneum), connective tissue and the surface of the organs were examined for encysted acanthocephalans. The lung was examined under a binocular microscope (Stemi 2000, Carl Zeiss Mikroskopie, Jena, 07740, Germany) for nematode infections. *Crenosoma striatum* and *Capillaria aerophila* from the bronchi and bronchioles were quantified. The stomach and the intestine were stored overnight in tap water in the refrigerator at 4°C to allow the intestinal parasites from the intestinal wall to move into the water. The next day, the water and the intestinal sections were examined under a binocular microscope (water with transmitted light, intestinal sections with direct light). All parasites found were identified to species after Beck & Pantchev [19] and quantified. Information about the taxonomic status, the habitat preference and the host specificity of the parasites found in this study are listed in Table S2.

All statistical analyses were conducted using IBM SPSS Statistics Version 20. To test for differences in parasite abundance between sex, age and species groups, a Mann-Whitney U-test was used. To test for differences in parasite prevalence between sex, age and species group we used a chi-square test.

## Results

In total, twelve parasite species were determined (Table 1). Ectoparasites included one flea species, the hedgehog flea *Archaeopsylla erinacei* and two tick species, the hedgehog tick *Ixodes hexagonus* and the castor bean tick *I. ricinus*. The lungworm *C. striatum* and *C. aerophila* were found in the lungs. In the intestines *Capillaria* spp., *Physaloptera clausa* (only found in the stomach) and one unidentified nematode, the trematode *Brachylaemus erinacei*, the cestode *Hymenolepis erinacei* and the acanthocephalans *Nephridiorhynchus major* and *Plagiorhynchus cylindraceus* were found.

**Table 1:** Macroparasite prevalences, abundances and ranges of dissected hedgehogs from the Czech Republic.

Parasites	Hedgehog species	Age	N	Prevalence %	P prevalence	Abundance (SD)	P abundance	Range
*Archaeopsylla erinacei*	*EE*		72	5.6		0.3 (1.5)		0–9
	*ER*		25	0		0		
*Ixodes hexagonus*	*EE*		72	8.3		0.3 (1.1)		0–7
	*ER*		25	8.0		0.8 (3.3)		0–16
*I. ricinus*	*EE*		72	0		0		
	*ER*		25	4.0		0.6 (3.2)		0–16
*Capillaria aerophila*	*EE*		71	26.4	0.173	3.3 (13.8)	0.163	0–112
	*ER*		25	12.0		0.7 (2.2)		0–10
*Capillaria* spp.	*EE*		72	75.0	0.309	261.9 (379.6)	**0.026**	0–1498
	*ER*		25	64.0		65.4 (215.4)		0–1083
*Crenosoma striatum*	*EE*	j	60	56.7	0.788	17.8 (37.0)	0.614	0–227
		a	11	25.0	0.617	1.2 (2.6)	0.229	0–7
	*ER*	j	18	50.0		9.3 (12.6)		0–36
		a	7	42.9		6.3 (10.3)		0–10
*Physaloptera clausa*	*EE*		72	2.8	**<0.001**	0.18 (1.31)	**<0.001**	0–11
	*ER*		25	36.0		9.4 (18.4)		0–76
Nematode	*EE*		71	4.2		1.0 (6. 6)	0.792	0–54
	*ER*		25	4.0		0.2 (0.8)		0–4
*Brachylaemus erinacei*	*EE*	j	60	65.0	**<0.001**	96.7 (198.3)	**<0.001**	0–1184
		a	12	25.0	0.236	3.7 (7.3)	0.162	0–22
	*ER*	j	18	16.7		0.2 (0.4)		0–1
		a	7	0		0 (0)		
*Hymenolepis erinacei*	*EE*	j	60	0	0.051	0 (0)	**0.009**	0
		a	12	8.3	1.0	0.7 (2.4)	0.804	0–8
	*ER*	j	18	11.1		3.4 (13.9)		0–59
		a	7	14.3		0.9 (2.3)		0–6
*Nephridiorhynchus major*	*EE*		71	4.2	**<0.001**	0.14 (1.1)	**<0.001**	0–9
	*ER*		25	40.0		4.9 (7.5)		0–23
*Plaghiorhynchus cylindraceus*	*EE*		72	5.6	0.198	0.2 (0.8)	0.09	0–5
	*ER*		25	16.0		1.6 (5.1)		0–24

Hedgehogs from different sexes and ages are pooled, except for *Crenosoma striatum*, *Brachylaemus erinacei* and *Hymenolepis erinacei* for which age groups were treated separately; *EE*  =  *Erinaceus europaeus*, *ER*  =  *Erinaceus roumanicus*, j =  juvenile, a =  adult, N =  number of samples, SD  =  standard deviation, hoglets (N = 8) are not represented in the table. Note: developmental stages of ticks were pooled. P =  probability based on χ^2^-test for differences in prevalence and Mann-Whitney U-test for differences in abundance, p-values represent the differences between the two hedgehog species, ecotparasite prevalence and abundance were not statistically analyzed, because the sampling method might lead to biases.

Since none of the hoglets were parasitized, they were eliminated from further statistical analysis. We did not find any significant differences in parasitization between the sexes. We therefore pooled the sexes in each age group. We could not find any differences between age groups for *ER*. For *EE* we found higher mean abundances of *C. striatum* (p = 0.01), *B. erinacei* (p = 0.006) and lower abundances of *H. erinacei* (p = 0.02) in juveniles compared to adult animals (Table 1). Therefore we treated hedgehog age groups for those parasites separately for further statistical analysis. For all other parasites the age groups were pooled.

When we compared the differences in mean parasite infection rates between the two hedgehog species (Mann-Whitney U-test) we found significant differences for *H. erinacei* from the intestines for juveniles (p = 0.009), *B. erinacei* for juveniles (p<0.001), *Capillaria* spp. (p = 0.026), *P. clausa* (p<0.001) and *N. major* (p<0.001). The infection rates with *B. erinacei* and *Capillaria* spp. were higher in *EE*, while *ER* showed higher infection rates with *H. erinacei*, *P. clausa* and *N. major* (Table 1). Similar results were found when we compared the prevalences of the parasite species between *EE* and *ER*. *EE* showed higher prevalences of *B. erinacei* in juveniles (χ^2^_1_ = 13.015, p<0.001), while *ER* had higher prevalences for *P. clausa* (χ^2^_1_ = 20.371, p<0.001) and *N. major* (χ^2^_1_ = 20.53, p<0.001).

## Discussion

Neutral and adaptive changes during parasite-host coevolution are likely to affect the population and community attributes in both the host and the parasite. In the host species, with demography and range history affected by Pleistocene climatic oscillations, parasites may undergo complicated evolution [20]. Neutral evolution might occur during refugial, peripatric isolation and recurrent bottlenecks during population re-expansions, including founder effects causing parasite release, genetic drift and other factors acting on small populations. Site-specific adaptive responses may occur as well. These may culminate in the extinction of particular parasite species in a particular host lineage or in allopatric speciation. Both would contribute to divergence of parasite faunas between sister host species. On the other hand, parasites may affect evolution of the host, for example via parasite-mediated selection acting in genes regulating immune defense, and may contribute to the host speciation process. Here we show that although there do not appear to be any major morphological changes in the species infesting the two hedgehog species in their zone of overlap, there are considerable differences in the prevalence and intensity of infestation by a variety of endoparasitic species. In order to determine how significant these changes are it is also necessary to consider completely allopatric population of both host species.

### Divergent patterns of parasite diversity and load

Many studies on the parasite fauna of *E. erinacei* were carried out in the late 1970s and 1980s (e.g. [21]–[24]). These are complimented by several more recent studies [16], [25]–[27]. In contrast to this, only limited information is available on the parasites of *EC* (e.g. [28]) and *ER* (e.g. [29]). With the exception of the work of [29] using fecal analysis to determine the helminth fauna of *EE* and *ER* in the contact zone in Poland, comparative studies for both species are lacking.

All parasites found in this study have been found previously in or on hedgehogs (e.g. [16], [21], [26], [30]–[32]), although molecular analysis will be needed to confirm that no sibling species are present. If available, when comparing this study to other studies hedgehog sample size (n) and mean abundances (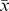) of the other studies will be provided in brackets.

Abundances and prevalences of the flea *A. erinacei* from this study are relatively low. Egli [33] reports prevalences from *EE* (n = 135) of 43.7% in Switzerland and Visser *et al.* [34] of 84.2% in Germany (n = 76). This also applies for *ER* from Hungary where Földvari *et al.* [35] found prevalences between 26.3% and 72.1% (n = 247). Beck & Clark [36] and Beck *et al.* [37] even state that every hedgehog is to a greater or lesser extent infested with the hedgehog flea.

Both *I. ricinus* and *I. hexagonus* are also frequently found on hedgehogs. As for the hedgehog flea, tick abundances and prevalence were lower in the present study compared to others. *Ixodes ricinus* prevalences on hedgehogs from Germany (23.4%, n = 133, 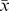 = 2.83 [16]) and Switzerland (11.1%, n = 135, [33]) were higher than 0% for *EE* and 4% for *ER* in the present study. For *I. hexagonus* Pfäffle [16] found prevalences of 53.3% (n = 133, 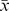 = 47.56) and 40% (n = 30, 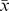 = 23.77) for *EE* from Germany and the UK, respectively. Egli [33] determined *I. hexagonus* prevalences of 58.5% for *EE* (n = 135). However, Földvari *et al.* [35] found *I. hexagonus* prevalences of only 1.1% on *ER* from Hungary (n = 247). They indicate that this low prevalence could be related to the high hedgehog density on Margaret Island where the study was conducted. Such high hedgehog population densities in semi-natural environments might lead to higher densities of *I. ricinus* compared to *I. hexagonus* [38]. Almost all of the animals from the present study came from wildlife rescue centers where they were treated for injuries or symptoms of disease. During this time both ticks and fleas might have been collected by the caretakers or left and dropped of the host, respectively. Therefore our data for ectoparasite prevalences and abundances might well be imprecise and not express the natural infestation rates of *EE* and *ER* in the Czech Republic. However, the results do provide an insight into the species of ectoparasites found on both Czech hedgehog species.

The lungworm *C. striatum* is specific to hedgehogs and the most important parasite of the lung [23], [39]. Depending on the habitat and the type of examination carried out (coprological vs. dissection), prevalences lie between 45% and 77.5% for *EE* ([16] dissection, n = 133, 66.4%, 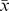 = 20.88; [23] coprological, n = 127, 52–72.3%; [24] dissection, n = 53, 66%; [40] dissection, n = 125, 62.4%, 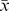 = 46.1; [41] coprological, n = 155, 77.5%). This is comparable with the results of the present study (25–56.7%, 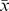 = 1.18–17.83). It seems that infection rates of *C. striatum* in *ER* are typically lower than in *EE*. Furmaga [30] found prevalences of 14.29% (dissection, n = 14) and Mizgajska *et al.* [29] found prevalences of 4.6% (coprological, n = 44). In the present study no significant differences between the infection rates of *C. striatum* of *EE* and *ER* were found and the intensity of infections probably do not depend on the hedgehog species but on the distribution of infected intermediate hosts (land snails), individual food preferences and immune status.

*Capillaria aerophila* is found in the smaller bronchi and is usually less abundant than the lungworm *C. striatum*, but can cause similar symptoms, such as weight loss, bronchitis and pulmonary damage [16], [24], [42]. The prevalences of *C. aerophila* found in *EE* from the present study are comparable to findings from other locations (Table 2). The prevalence of *C. aerophila* infections in *ER* is comparably lower than the prevalences found in the study of Mizgajska *et al.* [29]. However, it should be noted that the information about the parasite fauna of *ER* is very scarce.

**Table 2:** Comparison of the macroparasite fauna of *Erinaceus europaeus* from Central Europe (without the contact zones of *E. europaeus* and *E. roumanicus* in the Czech Republic and Poland) and its' southern European refuges.

Parasite species	Central Europe ^[16], [21], [23], [33], [41], [62]–[66]^	Spain ^[40], [53]^	Italy ^[50], [51], [54], [67]^
*Capillaria aerophila* (syn. *Eucoleus aerophilus*, incl. *C. tenuis*, syn. *E. tenuis*)	yes (19.4–69.6%)	yes (1.7%)	no
*Capillaria hepatica* (syn. *Calodium hepaticum*)	no	yes (0.8%)	no
*Capillaria* spp. (incl. *C. erinacei*, syn. *Aonchotheca erinacei*, *C. ovoreticulata*)	yes (37.1–89,6%)	yes (41.6%)	yes (30.7–66%)
*Crenosoma striatum*	yes (10.3–79.9%)	yes (62.4–83%)	yes (47–77%)
*Gongylonema* spp.	no	no	yes (43.5–50%)
*Haemonchus contortus*	no	no	yes (3%)
*Physaloptera clausa*	no	yes (6.4%)	yes (3%)
*Porrocaecum* sp.	no	yes (22.4%)	no
*Pterygodermatites plagiostoma*	no	yes (0.8%)	no
*Spirura rytipleurites seurati*	no	yes (21.6%)	yes (29–30.7%)
*Trichuris* spp.	yes (8%)	no	no
*Brachylaemus erinacei* (syn. *Brachylaima erinacei*)	yes (0.4–53.4%)	yes (38.4%)	yes (11–53%)
*Brachylecithum aetechini*	no	no	yes (2.5%)
*Dicrocoelium dendriticum*	no	no	yes (2.5%)
*Hymenolepis erinacei*	yes (0.2–4%)	no	no
*Mesocestoides* sp.	no	no	yes (3–7.6%)
*Plagiorhynchus cylindraceus*	yes (16.1%)	no	no
*Prosthorhynchus* spp.	no	yes (4%)	no
*Nephridiorhynchus major* (syn. *Nephridiacanthus major*)	no	yes (0.8%)	yes (23.5–69.2%)
Species richness	7	11	11

Note: ectoparasites were excluded from the comparison.

[16] Parasitological examination of 133 *E. europaeus* from Germany.

[21]: Parasitological examination of 410 *E. europaeus* from Germany.

[23] Coprological examination of 754 *E. europaeus* from Germany.

[33] Coprological examination of 135 *E. europaeus* from Switzerland.

[40] Parastiological examination of 125 *E. europaeus* from 26 provinces of the Iberian Peninsula, Spain.

[41] Coprological and parasitological examination of (numbers not known) *E. europaeus* from Richterswil, Switzerland.

[50] Parasitological examination of 39 *E. europaeus* from the provinces of Messina, Catania, Agrigento and Siracusa, Sicily, Italy.

[51] Parasitological examination of 126 *E. europaeus* from Sardinia (n = 34), Sicily (n = 39) and Emilia-Romagna (n = 53), Italy. (including data from [50] and [54]).

[53] Parasitological examination of the lungs and hearts of *E. europaeus* (n =  unknown) from Galicia, Spain.

[54] Parasitological examination of 34 *E. europaeus* from Sardinia, Italy.

[62] Parasitological examination of 175 *E. europaeus* from Switzerland (no prevalences available).

[63] Coprological examination of 212 *E. europaeus* from Pfaffenhoffen, Glonn and Munich, Germany.

[64] Parasitological examination of faeces (n = 601), guts (n = 232) and lungs (n = 209) of *E. europaeus* from East Germany (former GDR).

[65] Coprological examination of 243 *E. europaeus* from Germany.

[66] Coprological examination of 334 *E. europaeus* from Germany.

[67] Parasitological examination of one *E. europaeus* from Elba, Italy.

In addition to the findings from Edelenyi & Szabo [43], this is the first time that *P. cylindraceus* has been described for *ER*, although it has been described from the Czech Republic in *EE* by Prokopic [44] (1957, syn. *Prosthorhynchus jormosus*). It is an intestinal parasite of passerine birds which is sporadically found in the intestinal tracts of mammals, causing diarrhea, peritonitis and sometimes increased mortality [32]. Infections seem to occur more often in juveniles than in adult animals, since younger animals also feed on unpalatable prey like woodlice, which are the intermediate hosts for this parasite [32], [45]. However, we were not able to find significant differences in infection rates between adults and juveniles neither for *EE* nor for *ER*.

Cestode infections are rare for *EE*, prevalence is normally low and restricted to certain regions (e.g. [23] *EE*, n = 754, 0.39%; [31] *EE*, n = 39, 8%; [46] *EE*, n = 437, 0.7%). In *ER* and *EC* infections seem to be more common ([28] *EC*, n = 18, 55%; Pfäffle unpublished data). However, in the present study only one *EE* and three *ER* were infected with *H. erinacei*, while Mizgajska *et al.* [29] did not find any cestode infections at all. All three infected *ER* came from Prague, while the infected *EE* originated from Kocbere. These results indicate that the abundance of *H. erinacei* might also be restricted to certain regions in the Czech Republic. Nevertheless, the sample size might have been too small to support this hypothesis and it is not possible to draw conclusions as to whether the differences in infection rates are species dependent on or are influenced by other factors.

We found higher abundances and prevalences of *B. erinacei* in juvenile *EE* compared to juvenile *ER* and higher mean infection rates with *Capillaria* spp. in *EE* compared to *ER* in all age groups. Abundances and prevalence of *P. clausa* and *N. major* where higher in *ER* compared to *EE* (all age groups). *Capillaria* spp. are common parasites of hedgehogs and can reach high prevalence (up to 90%) and intensities (see [16], [25], [29]). *Capillaria* spp. can be transmitted directly or indirectly via the ingestion of earthworms. They can have a severe effect on the body condition of hedgehogs, which might be increased during periods of high stress, for example during the reproductive phase or hibernation, and higher hedgehog population densities might increase the transmission rates between individuals, hence increasing prevalences and abundances [16]. The work of Mizgajska *et al.* [29] on *Capillaria* spp. showed infections in *EE* and *ER* which were the opposite of those found in the present study, with *ER* having higher prevalences than *EE*. It seems that this parasite is neither specific for, nor occurs predominantly in, a certain hedgehog species and that there is a high variability in infection rates dependent on region and habitat.

*Brachylaemus* infections were both higher in prevalence and abundance in juvenile *EE* than in juvenile *ER*. This is comparable to the study by Mizgajska *et al.* [29], where only *EE* (n = 15, 33%) were infected with trematodes. *Brachylaemus erinacei* is host specific [46], transmitted via the ingestion of various intermediate gastropod hosts [47] and can cause diarrhea, hemorrhagic enteritis, inflammation of the bile ducts, anemia and death [39], [42], [48].

Both *P. clausa* and *N. major* are uncommon in *EE* but occur in both Eastern European hedgehog species ([28] *EC*, n = 41, *P. clausa*: 72.2%, *N. major*: 50%; [29] *ER*, n = 44, *P. clausa*: 13.6%; [30] *ER*, n = 14, *P. clausa*: 28.57, *N. major*: 7.14%; [49] *EC*, n = 11, *N. major*: 63.64%). Studies from southern Europe also found those parasites in *EE* ([40] Iberian Peninsula, Spain, n = 125, *P. clausa*: 6.4%, *N. major*: 0.8%; [50] Sicily, Italy, n = 39, *N. major*: 69.2%; [51] Sicily, Sardinia, Emilia-Romagna, Italy, n =  34–53, *P. clausa*: 0–3%, *N. major*: 0–69%). However, more recent studies did not find either of these species in *EE* from Central Europe and the UK [16], [25], [27]. Both parasite species are transmitted via the ingestion of infected insect intermediate hosts [52].

In general, the parasite fauna of the best studied species, *EE*, is relatively consistent throughout its range, although data from Spain [40], [53] and Italy [50], [51], [54] suggest that at least within these refuges there is a slightly higher parasite species diversity than further north (Table 2).

### Evolutionary interpretations

A detailed comparison of the ecology of host both species is still missing. The existence of a relatively large zone of overlap can be viewed as a natural, large scale, common-garden experiment, which could be utilized for studying the ecological diversification of both lineages with a similar environmental background. The landscape genetic analysis from central Europe points to differences in the altitudinal distribution of *EE* and *ER*, indicating at least some ecological differentiation [10]. However, both species can occur in similar habitats and even syntopically, in rural, suburban and urban habitats. *EE* and *ER* seem to be essentially similar in their feeding ecology (e.g. [55]–[61]). The diet consists mainly of a variety of invertebrates, usually with a few main prey types such as beetles, caterpillars, earthworms, slugs and snails, which can act as intermediate hosts of various parasite species [13]. However animals from different regions show their own particular spectrum of prey items [13]. The fact that all parasite species with significantly different abundances in *EE* and *ER* are intestinal forms nevertheless indicates possible diversification of trophic niches between these sister hedgehog species.

Although definite quantitative differences were found in prevalences and intensities of infection by certain parasite species between the two hedgehog species, qualitative differences in terms of differences in species composition were not apparent. However, as species identification was carried out morphologically, this does not exclude the possibility of cryptic variation in studied species. In order to determine the degree of divergence, and potentially introgression after secondary contact, a molecular study of the parasites should be carried out.

## Supporting Information

Table S1 **Origins from dissected hedgehog from the Czech Republic.**
(DOCX)

Table S2 **Taxonomic status, niche and host specificity of parasites found in the present study.**
(DOCX)

Dataset S1 **Raw data.**
(XLSX)
