# Supplementary material for: Different Parasite Faunas in Sympatric Populations of Sister Hedgehog Species in a Secondary Contact Zone
Source: PLoS One. 2014 Dec 3;9(12):e114030. doi: 10.1371/journal.pone.0114030 (PMC4254975; doi:10.1371/journal.pone.0114030)
Supplement: Table S1 — Origins from dissected hedgehog from the Czech Republic. (DOCX) [file pone.0114030.s001.docx]

Table S1: Origins from dissected hedgehog from the Czech Republic. *ER* = *Erinaceus roumanicus*, *EE* = *E. europaeus*. Type of specimens are marked as RC – died in rescue center or RK – found as a roadkill

| Location | *ER* | *EE* | Total | Specimen | Coordinates |
| --- | --- | --- | --- | --- | --- |
| Beroun | 0 | 1 | 1 | RC |  |
| Bruntal | 0 | 10 | 10 | RC |  |
| Ceska Lipa | 0 | 2 | 2 | RC |  |
| Decin | 0 | 1 | 1 | RC |  |
| Doksy | 0 | 1 | 1 | RC |  |
| Jaromer (Nachod) | 0 | 2 | 2 | RC |  |
| Kocbere | 0 | 1 | 1 | RC |  |
| Kolin | 1 | 0 | 1 | RC |  |
| Litomerice | 4 | 5 | 9 | RC |  |
| Louny | 0 | 1 | 1 | RC |  |
| Mlada Boleslav | 0 | 1 | 1 | RK |  |
| Most | 0 | 1 | 1 | RC |  |
| Novy Bor | 0 | 1 | 1 | RC |  |
| Petrovice | 1 | 0 | 1 | RK | 50.1679 N 16.0452 E |
| Prague | 17 | 7 | 24 | RC |  |
| Roudnice nad Labem | 0 | 2 | 2 | RC |  |
| Skalice | 1 | 0 | 1 | RK | 50.2853 N 15.8798 E |
| Steti | 0 | 1 | 1 | RC |  |
| Trutnov | 0 | 2 | 2 | RC |  |
| Vlasim | 1 | 40 | 41 | RC |  |
| Vlkov | 1 | 0 | 1 | RK | 50.3134 N 15.9004 E |
