# Supplementary material for: Different Parasite Faunas in Sympatric Populations of Sister Hedgehog Species in a Secondary Contact Zone
Source: PLoS One. 2014 Dec 3;9(12):e114030. doi: 10.1371/journal.pone.0114030 (PMC4254975; doi:10.1371/journal.pone.0114030)
Supplement: Table S2 — Taxonomic status, niche and host specificity of parasites found in the present study. (DOCX) [file pone.0114030.s002.docx]

Table S2: Taxonomic status, niche and host specificity of parasites found in the present study

| Parasites | Phylum | Order | Family | Niche | Hosts |
| --- | --- | --- | --- | --- | --- |
| *Archaeopsylla erinacei* | Arthropoda | Siphonaptera | Pulicidae | Body surface | Predominantly hedgehogs |
| *Ixodes hexagonus* | Arthropoda | Ixodida | Ixodidae | Body surface | Predominantly hedgehogs, canids |
| *I. ricinus* | Arthropoda | Ixodida | Ixodidae | Body surface | Reptiles, birds, mammals |
| *Capillaria aerophila* | Nematoda | Trichurida | Capillariidae | Smaller bronchi | Foxes, hedgehogs, carnivores |
| *Capillaria* spp. | Nematoda | Trichurida | Capillariidae | Intestinal tract | Predominantly hedgehogs, carnivores |
| *Crenosoma striatum* | Nematoda | Strongylida | Crenosomatidae | Bronchi | Hedgehogs |
| *Physaloptera clausa* | Nematoda | Spirurida | Physalopteridae | Stomach | Hedgehogs |
| *Brachylaemus erinacei* | Platyhelminthes | Strigeidida | Brachylaimidae | Intestinal tract, bile ducts | Hedgehogs |
| *Hymenolepis erinacei* | Platyhelminthes | Cyclophyllidea | Hymenolepididae | Intestinal tract | Hedgehogs |
| *Nephridiorhynchus major* | Acantocephala | Oligacanthorhynchida | Oligacanthorhynchidae | Intestinal tract | Hedgehogs |
| *Plaghiorhynchus cylindraceus* | Acantocephala | Polymorphida | Plagiorhynchidae | Intestinal tract, body cavity | Passerine birds, hedgehogs dead end hosts |
